# An Invertible Dynamic Graph Convolutional Network for Multi-Center ASD Classification

**DOI:** 10.3389/fnins.2021.828512

**Published:** 2022-02-04

**Authors:** Yueying Chen, Aiping Liu, Xueyang Fu, Jie Wen, Xun Chen

**Affiliations:** ^1^School of Information Science and Technology, University of Science and Technology of China, Hefei, China; ^2^USTC IAT-Huami Joint Laboratory for Brain-Machine Intelligence, Institute of Advanced Technology, University of Science and Technology of China, Hefei, China; ^3^Division of Life Sciences and Medicine, Department of Radiology, The First Affiliated Hospital of USTC (Anhui Provincial Hospital), University of Science and Technology of China, Hefei, China

**Keywords:** fMRI, graph convolutional networks, invertible networks, brain connectivity networks, autism spectrum disorder, disease classification

## Abstract

Autism Spectrum Disorder (ASD) is one common developmental disorder with great variations in symptoms and severity, making the diagnosis of ASD a challenging task. Existing deep learning models using brain connectivity features to classify ASD still suffer from degraded performance for multi-center data due to limited feature representation ability and insufficient interpretability. Given that Graph Convolutional Network (GCN) has demonstrated superiority in learning discriminative representations of brain connectivity networks, in this paper, we propose an invertible dynamic GCN model to identify ASD and investigate the alterations of connectivity patterns associated with the disease. In order to select explainable features from the model, invertible blocks are introduced in the whole network, and we are able to reconstruct the input dynamic features from the network's output. A pre-screening of connectivity features is adopted to reduce the redundancy of the input information, and a fully-connected layer is added to perform classification. The experimental results on 867 subjects show that our proposed method achieves superior disease classification performance. It provides an interpretable deep learning model for brain connectivity analysis and is of great potential in studying brain-related disorders.

## 1. Introduction

As one of the most common neurodevelopmental disorders, the exact etiology of Autism Spectrum Disorder (ASD) remains unknown. In the past 50 years, ASD has gone from a narrowly defined, rare disorder of childhood to a well-publicized disease, and recognized as a very common and heritable brain disorder. The major characteristic of ASD is being deficit in social interaction and social communication with repetitive and unusual behaviors and activities (Lord et al., 2018). Despite medical progress, the diagnosis of ASD still depends on the symptom-based clinical criteria with complex diagnostic steps. However, with increasing recognition of the importance of early diagnosis for effective intervention, more effort has been made on exploring other possible modalities and biomarkers for ASD identification.

With the development of neuroimaging technologies, resting-state functional Magnetic Resonance Imaging (rs-fMRI) has attracted increasing interest in ASD studies, which enjoys advantages of superior spatial resolution to accurately locate the active areas in the whole brain, overcoming the limitations of earlier tools such as positron emission tomography (PET), electroencephalography (EEG), and magnetoencephalography (MEG). By computing the correlation between fMRI time series of different regions of interests (ROIs), we can construct a functional connectivity network and many disorders may lead to the alterations in it (Li et al., 2016; Miller et al., 2016; Bachmann et al., 2018; Chandra et al., 2019; Zhang et al., 2020). For example, a widespread decrease of functional connectivity strengths was reported in patients with Alzheimer's Disease (AD) (Demirtaş et al., 2017). Studies showed that regional connectivity changes (both increase and decrease) of dopaminergic cortico-striatal and mesolimbic-striatal loops have been found in PD subjects (Filippi et al., 2018). ASD has also been suggested to be related to altered brain connectivity in the development of disease and has been extensively investigated (Kleinhans et al., 2008; Monk et al., 2009; Yerys et al., 2015; Dajani and Uddin, 2016; Xu et al., 2020). While a wide range of connectivity changes are reported, inconsistent conclusions have been observed in studies of functional connectivity in ASD, indicating the importance to thoroughly investigate the connectivity patterns with a large population of ASD.

Based on brain connectivity networks, machine learning, especially deep learning methods have further provided powerful tools to extract representative features associated with ASD and have greatly deepened our understanding of the disease (Chan et al., 2020). The classical machine learning techniques such as Support Vector Machines (SVM) are most widely used to identify patients from healthy controls in various studies (Subbaraju et al., 2017). For instance, Abraham et al. (2017) achieved 66.8% classification accuracy on 871 subjects obtained from ABIDE dataset.

Neural networks and deep learning methods such as autoencoder, Deep Neural Network (DNN) (Guo et al., 2017), Long Short Term Memory (LSTM) (Dvornek et al., 2018), and Convolutional Neural Network (CNN) (Haweel et al., 2021) have generated better performance in ASD classification. For instance, Yin et al. (2021) applied a DNN model and achieved the classification accuracy of 76.2% on 871 subjects of ABIDE dataset, and further improved the performance to an accuracy of 79.2% by combining DNN with an autoencoder.

Compared with traditional deep learning models, Graph Convolutional Network (GCN) can deal with data of non-Euclidean structure, which may be more suitable, and more interpretable for brain connectivity graph generated by fMRI. GCN has been used to classify ASD and select biomarkers from typical developing subjects (Ktena et al., 2018; Parisot et al., 2018). Recently, with a connectivity-based GCN model, 70.7% accuracy for classifying 1057 subjects (525 ASD and 532 healthy controls) has been reported (Wang et al., 2021). It's worth noting that when integrating information from more modalities, we may obtain higher classification accuracy. For instance, 85.06% of accuracy in ASD classification has been reported in Rakić et al. (2020) based on both structural MRI (sMRI) and fMRI features of 368 ASD and 449 healthy control subjects using an autoencoder model. While more modalities are beneficial to disease identification, it requires extra resources on data collection. In this paper, we are more interested in resting-state fMRI and focus on the ASD classification using brain connectivity features based on fMRI signals.

However, most deep learning models are limited in interpretation because of their black box representation. Although the classification performances of most deep learning networks are superior to those of traditional or interpretable methods, the features they finally generate can hardly be corresponded to the inputs, challenging the selection of helpful biomarkers. To overcome this shortcoming, Jacobsen et al. (2018) proposed an invertible network using a fully-connected layer as an inner trainable network, which can accurately reconstruct the inputs to a layer from its outputs without any degradation of classification accuracy. Given its superiority, Zhuang et al. (2019) proposed an invertible network for ASD classification, and gained 71% accuracy on the whole ABIDE dataset.

To improve the model interpretability and to better utilize structural, spatial, and temporal characteristics of brain connectivity networks, in this paper, we propose an invertible dynamic GCN (ID-GCN) model for ASD classification. More specifically, invertible blocks are utilized in the whole network, capable of reconstructing the input features from the output of the network, followed by a fully-connected layer to perform classification. Additionally, we select the connectivity features with a pre-screening operation to reduce the redundancy of the input information. The proposed method is verified on multi-center ABIDE datasets and the results demonstrate its effectiveness for disease classification and potential for studying the disease-related connectivity features. The contributions of this paper are summarized as:

- An invertible graph convolutional network is designed for disease classification based on brain connectivity networks. It is capable of generating disease-related interpretable connectivity features and improving classification accuracy.
- The proposed model integrates the structural, spatial, and dynamic information of the brain connectivity networks, and a prior selection of the features is adopted to reduce the redundancy of the input features.
- The proposed method has been validated on ABIDE dataset with superior performance.

## 2. Methods

In this section, we first provide the notations and their definitions used in this paper, then we introduce our proposed invertible dynamic GCN model in detail.

### 2.1. Notations and Definitions

In this paper, we use *G*(*V, E*) to represent a graph, where *V* = {*v*_1_, *v*_2_, …, *v*_*n*_} is the set of nodes, and *E* = {*e*_*ij*_} is the set of edges. In the spatial connectivity graph, *e*_*ij*_ represents the Euclidean distance of two connected nodes, and in the functional connectivity graph, *e*_*ij*_ represents their connectivity strength. Additionally, let *A* denote the adjacency matrix of the graph and *X* denote the correlation matrix, in which every row represents a node's features.

### 2.2. Graph Convolutional Network

Graph Convolutional Network is a deep learning architecture, which can not only use the data itself but also the relationship between data represented as a graph. Through the adjacency matrix *A* of the graph, we can first calculate the normalized Laplacian matrix of *X*, which calculation formula is:


(1) $$\begin{array}{l} L=I-D^{-\frac{1}{2}}AD^{-\frac{1}{2}} \end{array}$$

Where *I* is an identity matrix and *D* is the diagonal degree matrix of *X*. Then, we get an eigendecomposition of the Laplacian matrix, *L* = *UΛU*^*T*^, where *U* is a set of orthonormal eigenvectors, and Λ = diag(λ_0_, …, λ_*n*−1_) is the matrix's non-negative eigenvalues. Based on these formulas, we get the propagation rule of graph convolution layers is:


(2) $$\begin{array}{l} X^{l}=\sigma \left(U\Theta \left(\Lambda \right)U^{T}X^{l-1}\right) \end{array}$$

Where σ is the activation function of the layer, and Θ(·) is the GCN convolution kernel. To simplify the calculation, we then fit the kernel by Chebyshev polynomials of order *k* (Hammond et al., 2011), which can be derived from:


(3) $$\begin{array}{l} T_{k}\left(c\right)=2cT_{k-1}\left(c\right)-T_{k-2}\left(c\right) \end{array}$$

(4) $$\begin{array}{l} T_{0}\left(c\right)=I,T_{1}\left(c\right)=c \end{array}$$

And the fitting formula is:


(5) $$\begin{array}{l} \Theta \left(\Lambda \right)=\overset{K-1}{\underset{k=0}{\sum }}\beta_{k}T_{k}\left(\tilde{\Lambda }\right) \end{array}$$

(6) $$\begin{array}{l} \tilde{\Lambda }=\frac{2}{\lambda_{max}}\Lambda -I \end{array}$$

Where β_*k*_ is the weight coefficient of the *k*th Chebyshev polynomial, and λ_*max*_ is the max eigenvalue of the Laplacian matrix. Since the calculation of Chebyshev polynomials is performed only on eigenvectors Λ, it does not affect other matrix operations like doing eigendecomposition. So the Equation (2) can be expressed as:


(7) $$\begin{array}{l} X^{l}=\sigma \left(\left(\overset{K-1}{\underset{k=0}{\sum }}\beta_{k}T_{k}\left(\tilde{L}\right)\right)X^{l-1}\right) \end{array}$$

Where $\tilde{L}$ is defined as $\tilde{L}=\frac{2}{\lambda_{max}}L-I$. Then we substitute the trainable weight matrix *W* for β_*k*_, and get the final propagation rule of graph convolution layers as:


(8) $$\begin{array}{l} X^{l}=\sigma \left(\overset{K-1}{\underset{k=0}{\sum }}T_{k}\left(\tilde{L}\right)\left(X^{l-1}\right)W\right) \end{array}$$

### 2.3. Invertible Block

**Figure 1:**
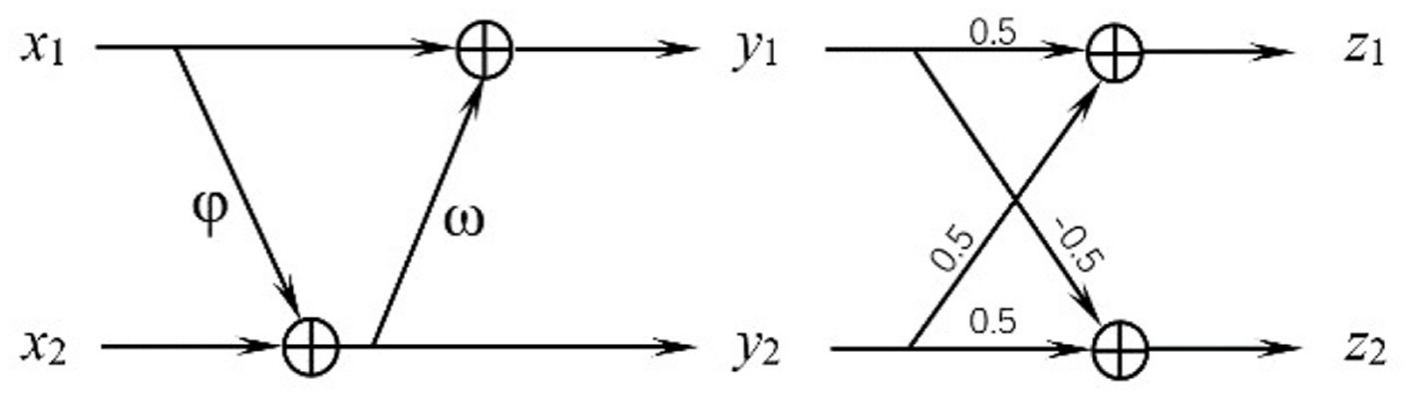
Structure of the invertible block.

The architecture of the invertible block is shown in Figure 1, where the inputs are *x*_1_ and *x*_2_, and the outputs are denoted as *z*_1_ and *z*_2_. Those feature maps have the same shape, and φ and ω can be defined as any functions. In this model, we define φ and ω as independent GCN modules using different graphs as their inputs, which will be introduced in detail in the next section. In order to fully blend the advantages of the two GCN modules, the outputs of the first block *y*_1_ and *y*_2_, are then calculated to their average and half of their difference as *z*_1_ and *z*_2_. This invertible block can reconstruct the input from its output, where the forward pass and inverse are:


(9) $$\{\begin{array}{l} y_{1}=x_{1}+\varphi (x_{2})\\ y_{2}=x_{2}+\omega (y_{1}) \end{array}\;\{\begin{array}{l} z_{1}=0.5(y_{1}+y_{2})\\ z_{2}=0.5(y_{2}-y_{1}) \end{array}$$

(10) $$\{\begin{array}{l} x_{2}=y_{2}-\omega (y_{1})\\ x_{1}=y_{1}-\varphi (x_{2}) \end{array}\;\{\begin{array}{l} y_{1}=z_{1}-z_{2}\\ y_{2}=z_{1}+z_{2} \end{array}$$

### 2.4. Invertible Dynamic GCN

**Figure 2:**
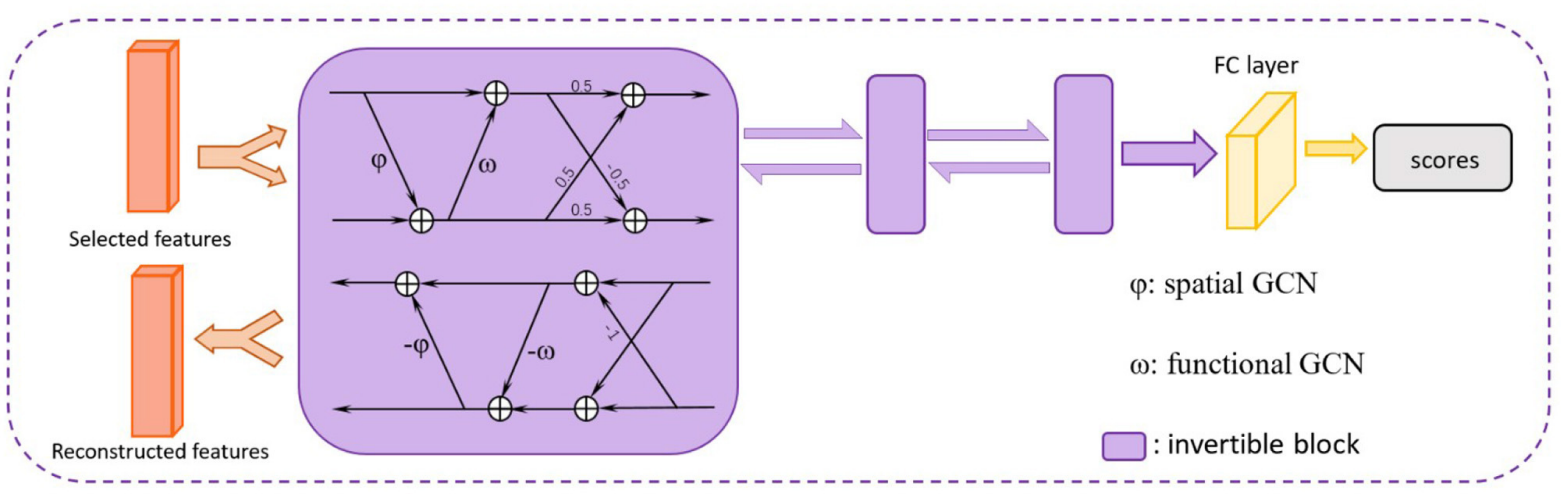
The proposed ID-GCN architecture. The selected features are trained in three invertible blocks. A fully connected (FC) layer is finally used to obtain the output scores for ASD classification. The whole network is reversible before the FC layer, meaning that we can reconstruct the informative disease-related brain connectivity patterns by selecting important output features of the network.

In order to incorporate additional spatial and temporal characteristics of the brain functional connectivity network constructed by rs-fMRI data with better interpretability, we propose an invertible dynamic GCN (ID-GCN) model, which uses two different GCN as the function φ and ω in the invertible blocks to encode the functional connectivity graph and spatial connectivity graph of samples, respectively. The functional GCN, i.e., ω in the invertible block, uses the functional graph of each subject obtained by the correlation matrix. Meanwhile, the skeleton of the spatial graph is calculated directly according to the spatial distance between ROIs, and the connection weights are their correlation values. It is represented as φ for spatial GCN. The whole model includes three invertible blocks to extract explainable high dimensional features, and the inputs *x*_1_ and *x*_2_ of the first block are the same features that we send into the model. The proposed ID-GCN architecture for disease classification in this work is demonstrated in Figure 2.

To improve the computational efficiency and simplify the training process, for each node, the *k* connected nodes with the largest Pearson correlation coefficients in the functional graph or the smallest distance in the spatial graph are retained to construct a *k*-nearest graph. The correlation coefficients between each node and all other nodes are used as the sample's features which serve as input into the ID-GCN model. A fully connected layer with softmax is applied to perform the classification and the source of the collection site is included as an additional covariate. The cross-entropy is adopted in this model as loss function as:


(11) $$\begin{array}{l} L=\frac{1}{N}\underset{i}{\sum }-y_{i}*log\left(\hat{y_{i}}\right)-\left(1-y_{i}\right)*log\left(1-\hat{y_{i}}\right) \end{array}$$

where *y*_*i*_ is the label of the *i*th subject, $\hat{y_{i}}$ is the output of the network, and *N* is the number of subjects we use.

While there are usually hundreds of ROIs defined from the atlas, for a certain disease, it usually involves the changes of a portion of brain regions. Additionally, with a great individual variance of connectivity patterns, a large number of connectivity features may be easily disturbed by noise, affecting subsequent analysis and interpretation. However, reducing the number of ROIs in the input model may inevitably cause the loss of information. Therefore, rather than reducing the entire number of ROIs, we reduce the dimension of the input features of each ROI individually by selecting the *M* most important features for disease classification using random forest.

**Figure 3:**
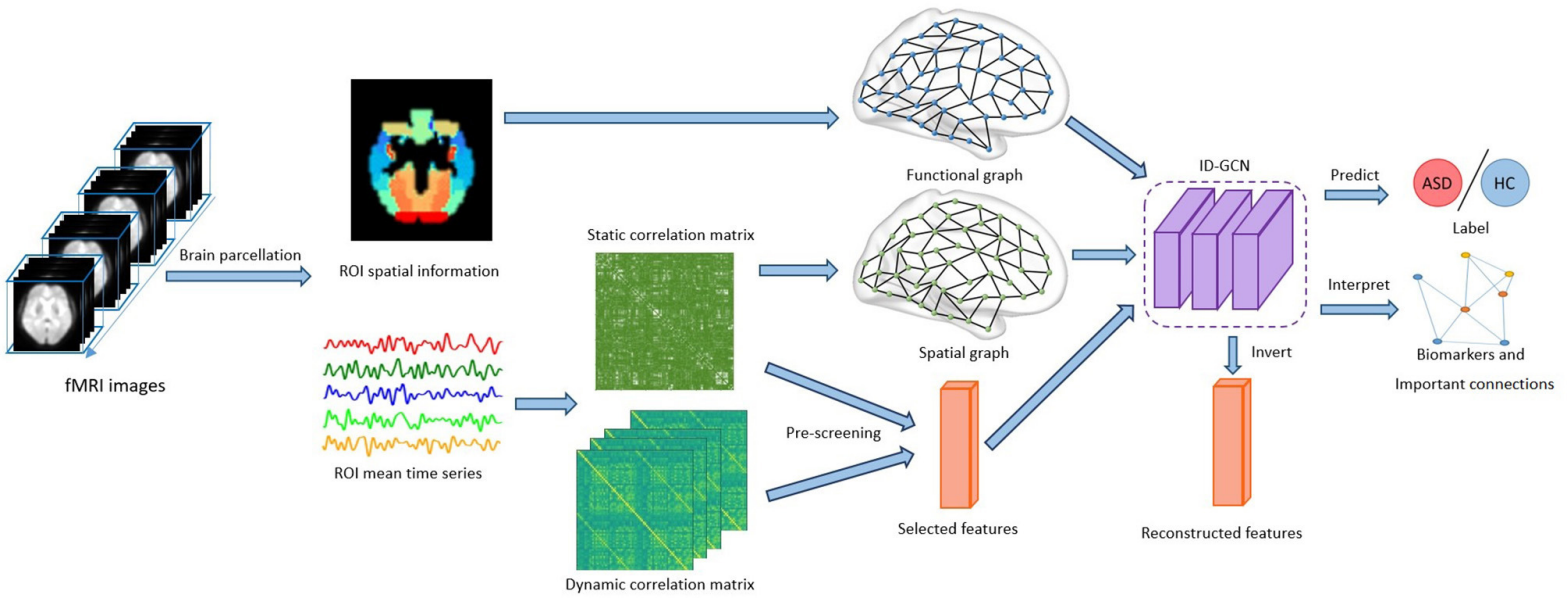
Overview of the proposed framework. The brain connectivity features inferred from the fMRI time series and brain parcellation are fed to the model. After training using the ID-GCN model, we obtain the predictions for ASD classification, and important brain connectivity features are selected accordingly.

As our brains are a dynamic system, time-varying connectivity features have been suggested to be related to the functioning of our brain. Thus, in this model, we further utilize the dynamics of connectivity as additional features for ASD classification. The time sliding window is applied to sample the time-dependent signals and get the correlation matrix *X*_*t*_ of each time window. The temporal variations of dynamic connectivity are then calculated as the auxiliary feature represented as *Ft*, which is concatenated with other connectivity features. After the pre-selection of random forest, the reserved feature matrix {*F*_*t*_} is combined with the selected feature *F* of the original correlation matrix *X* as the final input features. The overview of the proposed model is shown in Figure 3.

## 3. Experiments and Results

### 3.1. Real Dataset and Experimental Setting

We validated the proposed method on the publicly available ABIDE dataset (Martino et al., 2014), and chose 416 ASD subjects and 451 healthy controls (HC) from 13 acquisition sites. The phenotypical information of each acquisition site can refer to Table 1. The dataset was preprocessed with the Configurable Pipeline for the Analysis of Connectomes (C-PAC) (Sikka et al., 2013), which includes skull striping, slice timing correction, motion correction, global mean intensity normalization, nuisance signal regression, and band-pass filtering (0.01–0.1 Hz). The fMRI images were registered to the standard anatomical space (MNI152). To define brain areas, the Harvard Oxford (HO) atlas was chosen, consisting of 110 ROIs. More details of the dataset may refer to ABIDE Preprocessed.

**Table 1:** Phenotypical information summary of ABIDE data.

**Site**	**ASD**	**HC**	**Gender**	**Total**	**Age**
			**(M/F)**		**(mean±std)**
PITT	30	27	49/8	57	18.9 ± 6.8
TRINITY	24	25	49/0	49	17.2 ± 3.6
UM_1	55	55	84/26	110	13.4 ± 2.9
UM_2	13	22	33/2	35	16 ± 3.3
USM	58	43	101/0	101	22.1 ± 7.6
YALE	28	28	40/16	56	12.7 ± 2.9
LEUVEN_1	14	15	29/0	29	22.6 ± 3.5
LEUVEN_2	15	20	27/8	35	14.2 ± 1.4
KKI	22	33	42/13	55	10.1 ± 1.3
NYU	79	105	147/37	184	15.3 ± 6.6
UCLA_1	41	32	63/10	73	13.2 ± 2.4
UCLA_2	13	13	24/2	26	12.5 ± 1.5
MAX_MUN	24	33	50/7	57	26.2 ± 11.9
TOTAL	416	451	738/129	867	16.4 ± 7.1

We implemented the proposed model in a 5-fold cross-validation setting, using 80% of the data for training and 20% for testing. We set the pre-selected feature number *M* as 48, combined with *J*= 10 auxiliary dynamic features. Additionally, the Chebyshev polynomial order was chosen as 3, and *k*= 3 nearest nodes were selected to generate our graphs.

To test the proposed method, we compared it with other methods including siamese GCN (Ktena et al., 2018), Random Forest, SVM, and GCN, evaluating its performance improvement induced by the combination of spatial and dynamic connectivity features, and testing the effectiveness of pre-screening on the features. In these models for comparison, features input in siamese GCN is the paired subject features as implemented in the study (Ktena et al., 2018), while the other models use the whole connectivity matrix of a single subject as inputs. All the methods were evaluated in terms of accuracy, AUC value, precision, recall, and F1-score. The definitions of them are as follows:


(12) $$\begin{array}{l} Accuracy=\left(TP+TN\right)/n \end{array}$$

(13) $$\begin{array}{l} Precision=TP/\left(TP+FP\right) \end{array}$$

(14) $$\begin{array}{l} Recall=TP/\left(TP+FN\right) \end{array}$$

(15) $$\begin{array}{l} F1-score=2*Precision*Recall/\left(Precision+Recall\right) \end{array}$$

where *n* is the total number of our subject, TP is true positive subject's number, TN is true negative, FP is false positive, FN denotes false negative, and AUC means the area under the ROC curve. We additionally performed ablation experiments to demonstrate the effects of each step of our method, including (1) GCN using the functional graph as input (GCN); (2) GCN using the spatial and functional graph in different layers (GCN adding spatial information); (3) ID-GCN with principal component analysis (PCA) for feature selection (ID-GCN with PCA); and (4) ID-GCN without dynamic features.

## 4. Results

**Figure 4:**
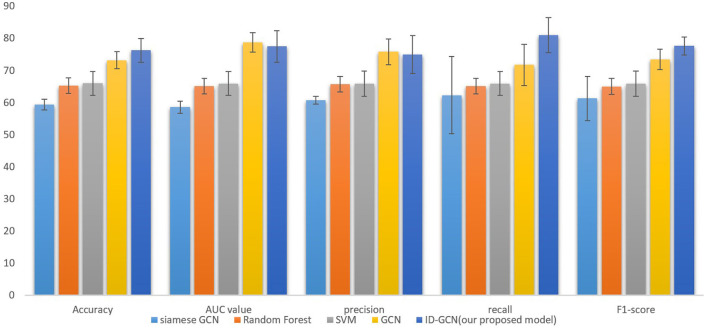
Comparison with traditional and GCN models including siamese GCN (Ktena et al., 2018), Random Forest, SVM, and GCN.

The classification results are shown in Figure 4 and Table 2. It's noted that our proposed model, ID-GCN achieves the highest classification accuracy as 76.3%. Specifically, our model demonstrates great improvement in all the evaluation metrics compared with traditional SVM and Random Forest models and obtains 3.1% gains in accuracy compared with GCN using the same hyperparameters. Siamese GCN used paired subject features as input and generated classification results by multiplying two feature matrices from shared weight GCN. However, it's noticed that siamese GCN demonstrated worse performance on the given dataset where the paired features didn't successfully distinguish the subjects in this case.

**Table 2:** Comparisons of different methods.

**Model**	**Accuracy**	**AUC**	**Precision**	**Recall**	**F1-score**
SVM	66.0 ± 3.7%	65.9 ± 3.7%	65.9 ± 3.9%	65.9 ± 3.7%	65.9 ± 3.9%
Random forest	65.3 ± 2.4%	65.1 ± 2.4%	65.7 ± 2.4%	65.1 ± 2.4%	65.0 ± 2.5%
GCN	73.2 ± 2.7%	**78.7**±3.0%	**75.8**±4.0%	71.7 ± 6.5%	73.4 ± 3.2%
Siamises GCN	59.4 ± 1.7%	58.6 ± 1.9%	60.7 ± 1.2%	62.3 ± 12.0%	61.3 ± 6.9%
ID-GCN(our model)	**76.3**±3.7%	77.5 ± 4.9%	75 ± 5.9%	**81.0**±5.5%	**77.6**±2.8%

Considering that the classification performance depends on the number of subjects, in order to have a fair comparison, we have tested our algorithm on the different number of subjects and show comparison with other state-of-the-art methods in Table 3. More specifically, we chose the number of subjects as 95, 459, 867, and 1,066, respectively. As they were examined on a different number of subjects, we didn't repeat their experiments but reported their datasets and results, only using same order of magnitude of subjects to run our model for better comparison. It can be seen that our results outperform other methods on the same order of magnitude of data. It's worth noting that with data from different centers, the accuracy may vary. As demonstrated in Table 3, we can notice that more subjects do not guarantee better performance which is partially due to the great inter-center and inter-subject variability. When using 95 subjects from the same acquisition center, both (Li et al., 2018) and our method achieve high classification accuracy, and our proposed method obtains better classification performance compared with that of Li et al. (2018). Furthermore, the model performance of every single center is provided in Table 4 that we train all the subjects and test the proposed method for each center separately. It shows that the classification accuracy varies across the centers, indicating great inter-center variability.

**Table 3:** Comparison with other SOTA methods.

**Model**	**Number of subjects**	**Accuracy**
DNN (Li et al., 2018)	95	85.3%
Combined MCNNEs (Aghdam et al., 2019)	459	70.45%
CNN-EW (Xing et al., 2018)	1096	66.88%
ASD-DiagNet (Eslami et al., 2019)	1035	70.1%
cGCN (Wang et al., 2021)	1057	70.7%
3D CNN (Thomas et al., 2020)	1162	64%
	95	**87.38** **%**
	459	**77.42** **%**
ID-GCN(our model)	867	**76.3** **%**
	1066	**71.44** **%**

**Table 4:** Model performance in each single center.

**Site**	**Number of subjects**	**Accuracy**
PITT	57	71.7 ± 6.7%
TRINITY	49	72.0 ± 11.7%
UM_1	110	75.5 ± 3.6%
UM_2	35	82.9 ± 10.7%
USM	101	84.8 ± 7.0%
YALE	56	80.0 ± 6.7%
LEUVEN_1	29	73.3 ± 8.3%
LEUVEN_2	35	74.3 ± 10.7%
KKI	55	74.5 ± 8.9%
NYU	184	76.2 ± 5.2%
UCLA_1	73	74.7 ± 8.8%
UCLA_2	26	83.3 ± 18.2%
MAX_MUN	57	66.6 ± 11.9%
TOTAL	867	76.3 ± 3.7%

Additionally, the studies with multimodality data often demonstrate better performances using the same method. For example, Rakić et al. (2020) gained 85.06% of accuracy using both sMRI and fMRI features in the classification of 817 subjects. However, in this paper, we focus on the functional connectivity features. Although the proposed method has improved the classification accuracy compared with other GCN models and has interpretability, it still has several limitations. The temporal variations of brain connectivity have been utilized to represent the dynamics of brain connectivity. However, it's unable to fully delineate the time-varying connectivity. The classification accuracy of our interpretable model is limited compared with some networks without interpretability. For better performance, RNN model with temporal connectivity networks will be explored in our future work. Additionally, the biological interpretation of the biomarkers selected from our invertible network has been limited investigated. The effective center-invariant biomarkers with sufficient biological meanings are warranted in future studies.

The results of ablation experiments are demonstrated in Table 5. It can be seen from the table that after adding spatial information as graph input, the accuracy of the model increased by over 1%, indicating the importance of the spatial information. As the number of connectivity features is large, great individual variation and noise may disturb the robust feature learning and degrade the classification performance. The feature selection, therefore, contributed to a significant improvement in the classification accuracy. We also evaluated other dimension reduction approach, i.e., Principal Component Analysis, for feature selection. As shown in Table 5, PCA led to less improvement in the classification accuracy. It may be due to the difficulty in the alignment of principal components across the subjects. Moreover, the temporal dynamics benefited the GCN model with a small accuracy gain.

**Table 5:** Ablation study on the effects of different components.

**Model**	**Accuracy**
GCN	73.2%
GCN adding spatial information	74.5%
ID-GCN with PCA	74.2%
ID-GCN without dynamic features	76.1%
ID-GCN(our model)	**76.3** **%**

**Figure 5:**
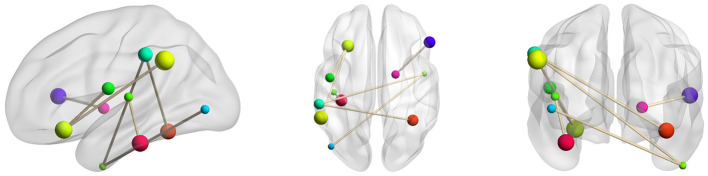
Selected key connectivity features for ASD classification.

**Figure 6:**
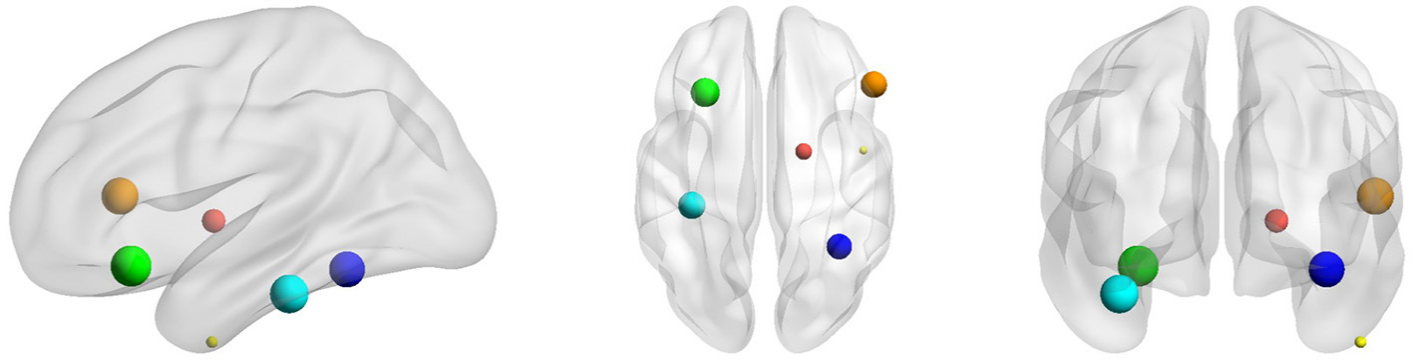
Selected key ROIs for ASD classification, including Right Pallidum (red), Right Inferior Frontal Gyrus (triangle part) (orange), Right Inferior Temporal Gyrus (anterior division) (yellow), Left Frontal Orbital Cortex (green), Left Temporal Fusiform Cortex (posterior division) (cyan), and Right Temporal Occipital Fusiform Cortex (blue).

In order to better understand ASD, we further identified the disease-related features by sorting the importance of each node's features extracted under the 5-fold cross-validation. The top 10% important connectivity edges were reconstructed as demonstrated in Figure 5 and Table 6. It's noted that the connections between Right Pallidum and Right Inferior Frontal Gyrus, Left Frontal Orbital Cortex and Left Central Opercular Cortex, and connections involving Left Supramarginal Gyrus and Right Inferior Temporal Gyrus greatly contributed to the classification accuracy. Additionally, we evaluated the impacts of nodes by excluding each node and examining its influence on classification performance. With such lesion operation, we were able to assess the importance of each node. As shown in Figure 6, the highly-rated ROIs include Right Pallidum, Right Inferior Frontal Gyrus (triangle part), Right Inferior Temporal Gyrus (anterior division), Left Frontal Orbital Cortex, Left Temporal Fusiform Cortex (posterior division), and Right Temporal Occipital Fusiform Cortex, indicating their potential ROIs for ASD.

**Table 6:** Important connectivity edges selected by feature reconstruction.

**ROI1**	**ROI2**
Right Pallidum	Right Inferior Frontal Gyrus
Left Frontal Orbital Cortex	Left Central Opercular Cortex
Left Temporal Fusiform Cortex (posterior division)	Left Heschl's Gyrus (includes H1 and H2)
Left Supramarginal Gyrus (anterior division)	Right Temporal Occipital Fusiform Cortex
Left Supramarginal Gyrus (posterior division)	Left Frontal Orbital Cortex
Right Inferior Temporal Gyrus (anterior division)	Left Supramarginal Gyrus (anterior division)
Right Inferior Temporal Gyrus (anterior division)	Left Lateral Occipital Cortex (inferior division)

## 5. Discussion and Conclusion

The early diagnosis of ASD is a challenging task as great variations exist in the symptoms. In addition to the clinical criterion, researchers have tried to identify the effective neuroimaging biomarkers for the better diagnosis of ASD. Brain connectivity features are promising for studying ASD as widespread connectivity changes have been observed in ASD. With various statistical and machine learning methods, we have largely expanded our understanding of the disease. However, the classification performance based on brain connectivity features is still limited, partially due to the insufficient representation ability for multi-center ASD data. It's, therefore, critical to learn the robust connectivity features for better representing the disease population. While the deep learning-based methods are promising, mos of them are designed in a black-box principle, challenging their biological interpretability.

In this study, we propose an explainable graph convolutional network, namely ID-GCN for multi-center ASD data classification and investigation by incorporating the functional, spatial and temporal information of the connectivity networks and using the invertible network to select interpretable biomarkers. The use of GCN aims to integrate the high-dimensional features of each node, and the invertible network is capable of reconstructing the extracted disease-related features back to the original connectivity graph. The proposed model contains two different GCN for brain functional connectivity and spatial connectivity, respectively. A random forest is adopted to narrow the feature space and reduce the redundancy of the data. We further integrate the dynamics of brain connectivity as important features for ASD classification. The experimental results on ABIDE dataset suggest the efficacy of our model. It is a potential classifier for large multi-center datasets despite their variations.

When classifying the ASD subjects, several connectivity features reconstructed by the model are assigned with higher importance. Those connections involve Right Pallidum, Right Inferior Frontal Gyrus, Left Frontal Orbital Cortex, Left Central Opercular Cortex, Left Temporal Fusiform Cortex, Right Temporal Occipital Fusiform Cortex, Left Supramarginal Gyrus and Right Inferior Temporal Gyrus, which are mostly consistent with the prior studies. For instance, the altered connectivity of Temporal Pole, Pallidum, and Frontal Orbital Cortex in ASD has been reported in Yerys et al. (2015); Dajani and Uddin (2016); Monk et al. (2009). In another line of studies, the changes of connectivity patterns in Fusiform Gyrus and Inferior Frontal Gyrus have been investigated for ASD subjects (Kleinhans et al., 2008; Xu et al., 2020). We additionally performed lesion analysis that sequentially removed each ROI and examined its impact on the classification accuracy. According to their contributions to the classification performance, eight ROIs including Right Superior Temporal Gyrus, Right Superior Frontal Gyrus, Right Pallidum, Right Inferior Frontal Gyrus (triangle part), Right Inferior Temporal Gyrus (anterior division), Left Frontal Orbital Cortex, Left Temporal Fusiform Cortex (posterior division), and Right Temporal Occipital Fusiform Cortex were chosen which are mostly involved in the connectivity features reconstructed by ID-GCN. It further substantiates the explainable features learned by the proposed method.

There are several parameters that need to be determined in the proposed model, and we have evaluated the impacts of different parameters on classification performance. Table 7 demonstrates the classification accuracy as a function of the numbers of neighbors. It's observed that classification performance depends on the values of *k*, and when *k*=3, we obtained the highest classification accuracy. It indicates that there may be only a few connected areas that are most robust across the subjects. We have also chosen the number of features *M* using the grid search in Table 8, and when *M*=48, it achieved the best performance. If the number of *M* is too small or too large, the performance of the model will decline greatly.

**Table 7:** The classification accuracy with different k.

**The value of k**	**2**	**3**	**4**	**5**	**6**	**8**	**10**	**15**	**20**
**Accuracy**	73.7%	**76.3** **%**	75.1%	76.0%	75.0%	75.0%	75.8%	74.7%	75.3%

**Table 8:** The classification accuracy with different M.

**The value of M**	**10**	**30**	**48**	**50**	**70**	**90**	**110**
**Accuracy**	72.1%	73.6%	**76.3** **%**	76.0%	75.7%	74.6%	73.9%

While the proposed method is capable to identify the disease-related features and achieves a competitive classification performance, it still has several limitations. The temporal variations of brain connectivity have been utilized to represent the dynamics of brain connectivity. However, it's unable to fully delineate the time-varying connectivity patterns, which can be further extended in our future work. The classification accuracy of our interpretable model is limited compared with some recent networks without interpretable modules. To further improve the performance, RNN models with temporal connectivity networks can be potential. Additionally, the biological interpretation of the biomarkers selected from our invertible network has been limited investigated. The effective center-invariant biomarkers with sufficient biological meanings are warranted in future studies.

## Data Availability Statement

The original contributions presented in the study are included in the article/supplementary material, further inquiries can be directed to the corresponding author/s.

## Author Contributions

YC, AL, and XF worked on the method and analyzed the data. JW interpreted the results. AL and XC supervised the project. The manuscript was drafted by YC and AL. All the authors have reviewed and revised the manuscript.

## Funding

This study was supported in part by the National Natural Science Foundation of China (Grants 61922075, 61701158 and 22077116), and in part by the USTC Research Funds of the Double First-Class Initiative (Grants YD2100002004 and YD9110002011).

## Conflict of Interest

The authors declare that the research was conducted in the absence of any commercial or financial relationships that could be construed as a potential conflict of interest.

## Publisher's Note

All claims expressed in this article are solely those of the authors and do not necessarily represent those of their affiliated organizations, or those of the publisher, the editors and the reviewers. Any product that may be evaluated in this article, or claim that may be made by its manufacturer, is not guaranteed or endorsed by the publisher.
